# Mothers’ oral health literacy and children’s oral health status in Pikine, Senegal: A pilot study

**DOI:** 10.1371/journal.pone.0226876

**Published:** 2020-01-23

**Authors:** Sérigne Dieng, Daouda Cisse, Pierre Lombrail, Sylvie Azogui-Lévy

**Affiliations:** 1 Educations and Health Practices Laboratory (LEPS EA 3412) Faculty of medicine, Paris 13 University, Bobigny, France; 2 Sanitary district of Koki, Louga, Sénégal; 3 Public Health Service, Department of Dentistry, Faculty of medicine, pharmacy and dentistry, University Cheik Anta Diop, Dakar, Sénégal; 4 Public Health Department, Faculty of medicine, Paris 13 University, Bobigny, France; 5 Dental Public Health Department, Paris Diderot University, Paris, France; Centre Hospitalier Regional Universitaire de Tours, FRANCE

## Abstract

**Context and objective:**

As elsewhere, disadvantaged children in Senegal are those most affected by dental diseases and difficulties in obtaining dental care. Studies conducted mainly in developed countries suggest that a low level of mothers’ OHL is correlated with poor oral health of their children. The objective of this study is to estimate the level of mothers’ OHL in Senegal and its relationto the dental health of their children.

**Methods:**

This cross-sectional epidemiological survey took place among 315 children aged from 3 to 9 years old and their mothers. It estimated the children’s dental health status by clinical examination which used a disposable examination kit and a headlamp, took place at the child’s home, in the mother’s presence. Examiners interviewing the mothers administered the Oral Health Literacy-Adult Questionnaire to determine their OHL and questioned them further about their social characteristics and their children’s dental health behaviour. Logistic regression and correlations were used for the statistical analysis.

**Results:**

The OHL score ranges from 0 to 17; mothers’ mean score was 6.5 (±3.1) and 56.5% had a low score (below the median). The prevalence of dental caries in children was 64.8%. Mothers’ high OHL is associated with children caries free and low prevalence of dental caries. The logistic regression showed a significantly protective relation between children’s dental caries and mothers’ high OHL scores (mean score 12–17) (OR = 0.51, 95% CI: 0.29–0.88), high educational level (OR = 0.42, 95% CI: 0.23–0.76) and a high level of social contact (OR = 0.31, 95% CI: 0.15–0.63). The structural analysis showed that OHL was significantly correlated with both the mothers’ social position (r = 0.61 and *P*<0.001) and the children’s caries (r = -0.26 and *P*<0.001).

**Conclusion:**

The OHL level of Senegalese mothers was significantly associated with their children’s dental caries. Improving mothers’ OHL might therefore help strengthen their capacities to promote oral health, thus helping to improve their children’s dental health and reduce inequalities.

## Introduction

The oral health status of children in Africa follows a social gradient like that of children in most areas of the world. Disadvantaged children have the largest share of oral diseases but do not have easy access to dental care [1]. The response of health services is mostly curative and generally inadequate due to the country’s insufficient resources. Although oral disease programmes, when they exist, improve the health of the whole population, their difficulty in reaching the most disadvantaged populations further aggravates health-related social inequalities [2]. In Senegal, the prevalence of dental caries is quite high among children [3]. A prevalence of 68% has been observed in children from 2 to 6 years old attending nursery schools in Pikine, a suburb of Dakar [4]. Several studies have shown that the social conditions in which children are raised and family psychosocial factors affect their oral health [5,6]. In this situation, it appears essential to develop primary prevention actions. Their effectiveness depends on mothers’ knowledge, attitudes, and practices (KAP) concerning oral health. The literature underlines the significant relation between the mothers’ oral health and that of their children [7], between mothers’ oral health skills and their attitudes toward their children’s oral health education (habits of brushing teeth, balanced diet) [8,9]. The attitudes linked to parents’ oral health are associated with the incidence of dental caries in young children [10]. Several conceptual models, all suggesting a multilevel approach, have been developed to analyse the factors associated with children’s oral health. The best known, that of Fisher-Owens et al (2007), depicts environmental contextual and societal relations in the form of a chain of elements [11]. Using this model, Duijster et al (2014) modelled the possible interrelations between the community, family, and individual factors linked to dental caries in children. The authors suggested that family determinants affecting children’s oral health are mediated by psychosocial factors that in turn have an effect on attitudes and behaviour relating to oral health, in particular, those of the mother [12].

Among the family characteristics, health literacy and its application to oral health, namely, oral health literacy (OHL), is a determinant that is associated with problems of access to prevention and oral healthcare [13]. Health literacy has three components: 1/ functional (ability to read and write); 2/ communicational/interactive (cognitive capacities to understand information); and 3/ critical (ability to critically analyse the information received anduse it to exercise increased control over life events) [14]. Thus OHL although sometimes defined mainly as literacy, is a social and individual resource that expresses the individual’s ability to obtain and process the basic oral health information required to take relevant oral health decisions [15,16].

Several studies have shown that mothers’ OHL levels are associated with their children’s oral health [8,17]. Mothers with a high OHL have children less affected by dental caries. Parents with low literacy generally have less knowledge of children’s oral health or preventive practices [18,19]. Several questionnaires have been formulated to estimate OHL. Some focus on reading and writing [20,21], while others try to assess literacy and evaluate oral health knowledge [22,23]. Sistani et al (2014) formulated an OHL indicator with 17 questions to estimate both dimensions [24]. It is quite short and appropriate for countries with few oral healthcare structures. No OHL questionnaire has previously been translated into French, and few studies have examined effect of OHL on oral health status and its role in shaping oral health inequalities in Africa. Our hypothesis was that the level of mothers’ OHL might determine their children’s oral health status and could therefore be used as a lever to improve the oral health of children in Senegal. As WHO has pointed out, Health literacy is a key strategy for promoting health, and appropriate strategies are essential to combat the causes of diseases [25].

The aim of this work is to estimate mothers’ level of OHL and study its relation to the oral health of children in a district of Dakar.

## Method

### Model of the study

We used WHO’s pathway model adapted for oral health [26] and the conceptual model of Fisher-Owens et al.[11] to build the model for our study. We have considered: 1/ structural determinants (socioeconomic context). 2/ intermediate determinants: social position of the parents (education level and occupation. 3/ direct determinants that means: material conditions of the household, psychosocial factors of the mothers, and knowledge, attitudes, practices and level of oral health literacy of the mothers. 4/ preventive behaviour and child’s oral health status.

### Study population

This cross-sectional study took place in the district of Pikine, a suburb of Dakar including 16 municipalities.

The study population comprised children from 3 to 9 years old and their mothers. The inclusion criteria required that each children had lived in the municipality chosen for at least one year and had lived with his or her mother from birth. A written informed consent had been signed by the mothers for their participation to study and that of their child. Children and mothers with a disability or disease liable to prevent data collection were excluded.

### Study variables

#### Mothers’demographic and social characteristics

The data collected included age (in two groups: 35 years or younger, and older than 35 and), level of education (in 2groups: one with no or only primary education, andthe other with a secondary or higher education), paid work status (working, not working), occupation (workers, intermediate professions, managers) and social support, estimated by the frequency of contacts with family, friendsand neighbours (less than once a month, at least once a month, and at least once a week).

#### Household characteristics

These are described by two variables: the estimated wealth of the household (based on a list of household goods) and household density (household members per room). A synthetic variable called “social position” was constructed from the data on education level, occupation and household density (see appendix).

#### Oral health literacy

This variable was estimated by the Oral Health Literacy-Adult Questionnaire (OHL-AQ) [24]. It contains 17 items, with a total score (non-weighted sum) ranging from 0 (lowest) to 17 (highest). For the statistical analyses, we divided the OHL into dichotomous (calculated from the median score) variables: low and high. We translated the questionnaire into the two primary languages in Pikine, Wolof and French (see appendix).

#### Mothers’ attitudes toward oral health and hygiene

The attitude to oral health was evaluated by a mini-questionnaire according to the importance given to 5 items [27]: limitation of sweet food consumption, use of fluoridated toothpaste, use of toothbrushes, regular visits to a dentist, keeping one’s mouth and teeth clean. Grouping the 4 possible responses to each item (not at all important, not important, important and very important) into pairs allowed us to classify the variable into 2 categories (negative attitude, positive attitude). Oral health practice was characterised by care practices (brushing with a toothbrush, with a natural toothbrush, or not at all).

### Outcome variable: Children’s dental health status

The outcome variable is evaluated by present or absent caries. Dental caries was measured by the prevalence in the sample; the number of children with at least one tooth decay were compared to the total number of children without tooth decay. Mothers reported their child’s tooth-brushing practices (yes, no).

### Data collection

The data on oral health status were collected from clinical examinations by the investigators, trained in WHO standards [28]. Interexaminer consistency estimated by the Kappa test was 87.2%. The examination, which used a disposable examination kit and a headlamp, took place at the child’s home, in the mother’spresence. The sociodemographic and OHL data were collected during a face-to-face interview conducted by the same trained examiner, using a questionnaire and conducted in French or Wolof. The questionnaire was pilot-tested beforehand on several mothers of families in a municipality not belonging to the sample. The survey took place from 16 March to 3 May 2015.

The National Committee of Health Research Ethics (CNERS) of the Senegal Ministry of Health and Social Action authorised the study.

### Analysis

Bivariate and multivariate analyzes were carried out. In the bivariate the chi-2 Pearson test with a *p* = 0.05 was used to derive the relationship between caries and independent variables. We use the bivariate analysis to select the variables for the multivariate analysis. Two logistic regression models are built to tested association between dental caries and OHL level with other socioeconomic factors. Structural equation type model (SEM) was also used to test the structure of the relations between the explanatory variables, OHL and dental caries. Results were analysed with STATA.13 software.

## Results

### Characteristics of children and mothers (Table 1)

**Table 1:** Characteristics of the study sample (N = 315).

Variables	Mean (sd)	N	%	Prevalence of caries	Non adjusted OR (CI 95%)	*P*
**Child’s age**	5 years (±7 months)					
3–6 years		199	63.2%	56.3%	1	
7–9 years		116	36.8%	79.3%	2.98(1.75–5.05)	<0.0001
**Child’s gender (N = 310)**	-					
Girl		173	56.5%	63.5%	1	
Boy		137	43.5%	66.4%	1.14(0.71–1.82)	<0.588
**Mother’s age**	34 years (±7 months)					
35 or younger		188	59.7%	66.5%	1	
Older than 35		127	40.3%	62.2%	0.83 (0.52–1.33)	<0.435
**Mother’s educational level**	-					
None or primary		215	68.3%	73%	1	
Secondary/ higher		100	31.7%	47%	0.33 (0.20–0.54)	<0.0001
**Mother doing paid work**	-					
No		151	47.9%	66.9%	1	
Yes		164	52.1%	62.8%	0.84(0.52–1.33)	<0.449
**Mother’s occupation (N = 314)**	-					
Worker		87	53.1%	70.1%	1	
Intermediate occupation		41	25%	63.4%	0.74 (0.34–1.62)	<0.027
Manager		36	21.9%	44.4%	0.34 (0.15–0.76)	
**Frequency of mother’s contacts (N = 314)**	-					
Fewer than one a month		54	21.6%	79.4%	1	<0.0001
At least one a month		83	36.8%	71.6%	0.65(0.31–1.33)	
At least one a week		67	41.6%	51.2%	0.27(0.14–0.54)	
**Wealth of household**	-					
Poorest		172	54.6%	73.8%	1	
Poor		95	30.2%	60%	0.53 (0.31–0.91)	<0.0001
Least poor		48	15.2%	41.7%	0.25 (0.13–0.49)	
**Household density**	-					
Denser		207	65.7%	69.1%	1	
Less dense		108	34.3%	56.5%	0.58 (0.36–0.94)	<0.027
**Child uses tooth brush**	-					
No		168	53.3%	84.5%	1	
Yes		147	46.7%	42.2%	0.33 (0.08–0.23	<0.001
**Mother’s oral hygiene practices**	-					
Natural toothbrush		136	43.2%	49.7%	1	
Toothbrush		179	56.8%	84.6%	0.18 (0.10–0.31)	<0.0001
**Mother’s attitude to oral health**	-					
Negative		173	54.9%	75.1%	1	
Positive		142	45.1%	52.1%	0.36(0.22–0.58)	<0.0001
**Mother’s OHL**	6.5 (±3.1)					
Low		178	56.5%	74.7%	1	
High		137	43.5%	51.8%	0.36 (0.22–0.58)	<0.0001
**Child dental caries**	2.5 (±2.7)					
No		111	35.2%	35.2%		
Yes		204	64.8%	64.8%		

N = number by category, % = percentage of subjects by category, p = significance threshold

In all, 315 children were examined and 315 mothers responded to the questionnaire. Six mothers refused to participate, giving as their reasons a lack of time or the father’s absence. The children’s average age was 5 years (±7 months); 63.2% were 3 to 6 years old, and therewere slightly more girls (56.5%) than boys. Mothers’ average age was 34 years (±7 months). Most mothers (68.3%) had a low educational level (none or primary only). More than half the mothers (52.1%) worked: 53.1% as manual workers, and 21.9% as managers. The mothers had a relatively active social life (41.6% had contacts with people in their environment at least once a week). The households were mostly poor (54.6%). The mean density of dwellings was three people per room, and it was higher than three for 65.7% of households.

Less than half (46.7%) of the mothers reported that their children brushed their teeth, 56.8% declared that they brushed their teeth and 45.1% considered oral health to be important. The mean OHL score of the mothers was 6.5 (±3.1) and the median score was 6; 56.5% of the mothers had a low OHL (and 43.5% a high OHL).

The prevalence of dental caries in the children was 64.8% and the mean mixed DMF index was 2.5 (±2.7). The prevalence of caries was significantly higher when the child was older, when the mother used a natural toothbrush, had a lower level of education, was a manual worker, and when the household was poorest (versus poor and least poor). OHL was also significantly associated with dental caries; a high OHL indicated protection: OR = 0.19, 95% CI: 0.07–0.49 (Table 1). OHL was significantly associated with tooth brushing, since mothers with a high OHL brushed their teeth more often, as did the children of such mothers (61.9%) (Table 2).

**Table 2:** Factors associated with mothers’ OHL.

Variables	Low OHL	High OHL	*P*
**Mother’s age group**			
20–35 yrs	109 (58%)	79 (42%)	<0.552
Over 35 yrs	69 (54.3%)	58 (45.7%)	
**Mother’s educational level**			
None or primary	158 (73.5%)	57 (26.5%)	<0.0001
Secondary or higher	20 (20%)	80 (80%)	
**Mother does paid work**			
No	104 (68.9%)	47 (31.1%)	<0.0001
Yes	74 (45.1%)	90 (54.9%)	
**Mother’s occupation (N = 314)**			
Worker	53 (60.9%)	34 (39.1%)	
Intermediate occupation	16 (39%)	25 (61%)	<0.0001
Manager	5 (13.9%)	31 (86.1%)	
**Frequency of mother’s contacts**			
Fewer than one a month	48(70.6%)	20 (29.4%)	
At least one a month	67(57.8%)	49 (42.2%)	<0.009
At least one a week	63(48.1%)	68(51.9%)	
**Mother’s attitude to oral health**			
Negative	137 (79.2%)	36 (20.8%)	<0.0001
Positive	41 (29.9%)	101 (71.1%)	
**Mother’s oral hygiene practices**			
Natural toothbrush	102 (75%)	34 (25%)	<0.0001
Toothbrush	76 (42.5%)	103 (57.5%)	
**Child uses tooth brush**			
No	122 (72.6%)	46 (27.4%)	<0.0001
Yes	56 (38.1%)	91 (61.9%)	
**Wealth of household**			
Poorest	129 (75%)	43 (25%)	<0.0001
Poor	39 (41.1%)	56 (58.9%)	
Least Poor	10 (20.8%)	38 (79.2%)	
**Household structure**			
Nuclear	39 (42.4%)	53 (57.6%)	<0.0001
Extended	131 (64.5%)	72 (35.5%)	
Others	8 (40%)	12 (60%)	
**Density of dwelling**			
Denser	141 (68.1%)	66 (31.9%)	<0.0001
Less dense	37(34.3%)	71 (65.7%)	

Furthermore, the bivariate analysis showed that a low OHL score was significantly associated with a low educational level (73.5% vs 20%), manual work (60.9% vs 39%), fewer than one contact a month, an unfavourable attitude to oral health and a poor household (Table 2).

The first logistic regression model showed that after adjustment for social position factors, mothers’ OHL (OR = 0.51, 95% CI: 0.29–0.88) and educational level (OR = 0.42, 95% CI: 0.23–0.76) were significantly associated with dental caries in children. In the second model, OHL (OR = 0.53, 95% CI: 0.31–0.91), household wealth (OR = 0.35, 95% CI: 0.17–0.74) and frequency of social contacts (OR = 0.31, 95% CI: 0.15–0.63) were significantly associated with dental caries in children: when the OHL was high, the household was (relatively) well-of the mother had active social support, and the child was significantly less prone to dental caries. When oral hygiene was integrated in model 2, with the KAP variable and the proxy measure of OHL, it was inversely associated (OR = 0.21, 95% CI 0.11–0.38) with the child’s dental caries, as was the frequency of the mother’s contacts (OR = 0.29, 95% CI: 0.14–0.62); household wealth, however, was only near the limit of significance with the 95% CI including unity (OR = 0.48, 95% CI: 0.23–1.01) (Table 3). Models 1 and 2 were validated by the Hosmer-Lemeshow test. In the structural equation model (SEM), mothers’ OHL was significantly associated with their social position (r = 0.61 and *P*<0.001) and children’s dental caries (r = -0.26 and *P*<0.001) (Table 4). The model fit is good, as shown by the RMSEA test: 0 at the lower bound; 0.0001 at the upper bound and *P* = 1 (thus >0.05).

**Table 3:** Factors associated with dental caries: Logistic regression analysis.

**Model 1**: caries, age, OHL and factors of the mother social position				
**Variables**	**Adjusted OR (CI 95%)**		**p**	
**Mothers’ age group**				
35 years or younger	1		<0.762	
Older than 35 years	0.93 (0.56–1.51)			
**Mother’s OHL**				
Low	1		**<0.016**	
High	0.51 (0.29–0.88)			
**Mother’s educational level**				
None or primary	1		**<0.004**	
Secondary or higher	0.42 (0.23–0.76)			
**Mother does paid work**				
No	1		<0.265	
Yes	0.74 (0.43–1.26)			
**Model 2**: caries, age, practice of oral hygiene, OHL and other direct determinants (household wealth and frequency of social contacts)				
**Variables**	**Adjusted OR (CI 95%)**	p	**Adjusted OR (CI 95%)**	p
**Mother’s age group**				
35 yrs or younger	1		1	
Over 35 yrs	0.83(0.49–1.42)	<0.505	0.94(0.57–1.58)	<0.836
**Frequency of mother’s contacts**				
Fewer than one a month	1		1	
At least one a month	0.68(0.32–1.49)	<0.340	0.69(0.32–1.45)	<0.323
At least one a week	0.29(0.14–0.62)	**<0.001**	0.31(0.15–0.63)	**<0.001**
**Household wealth**				
Poorest	1		1	
Poor	1.06(0.57–1.98)	<0.840	0.75(0.42–1.34)	<0.336
Least Poor	0.48(0.23–1.01)	**<0.055**	0.35(0.17–0.74)	**<0.006**
**Mother’s oral hygiene practices**				
Natural toothbrush	1		-	-
Toothbrush	0.21(0.11–0.38)	**<0.0001**		
**Mother’s OHL**				
Low	-	-	1	
High			0.53(0.31–0.91)	**<0.022**

**Table 4:** Relation between social position, mothers’ OHL and the dental caries of children: Structural analysis.

	Social position	p	Children’s dental caries	p
**Mother’s OHL**	r = 0.61	<0.001	r = -0.26	<0.001

## Discussion

The aim of this work was to estimate the relation of mothers’ OHL with their children’s oral health in Pikine, a district of Dakar. Our hypothesis was that these were likely to be associated.

We showed that disparities in dental caries exist as a function of both the mother’s socioeconomic characteristics (level of education, occupation and social support) and her OHL. These results agree with those of other studies [8,23], such as that by Vichayanrat et al. in Thailand, who found that a higher mother’s OHL was protective against children’s dental caries (OR = 0.87, 95% CI: 0.76–0.98) [23]. Our results thus confirm that a good OHL in mothers is beneficial for the oral health of children, probably because it indicates that they have learned about oral diseases and the means of preventing them and are able to both implement prevention and interact pertinently with the healthcare system [16,23]. The factors that determine the OHL level highlight the significant relation between OHL and educational level [29,30], level of wealth [31], and social support [32]. Our results agree with those in the literature. A minority of the mothers in our study (31.7%) had any secondary or higher education, and more than half the households were poor. Most of the mothers (78.4%) did have dynamic social support, however:communication and the sharing of information between the members of a network play an important role in determining a person’s knowledge, preventive behaviour, health status, and health service utilisation. Our results suggest a significant effect of social support. They may be explained by the country, Senegal, in which the study was carried out. Oral communication still plays a major role in healthcare relations. Using figurative language to clarify a message and emphasising one’s membership of the same cultural world facilitates the appropriation of knowledge and competencies [33].

Although causality cannot be asserted in a cross-sectional study, the analysis of multivariate models leads us to make two observations consistent with our data. First, mothers’ OHL is a direct determinant which, in its construction, interacts with the other direct determinants (material conditions and social support) and is affected by intermediate determinants. Indeed, OHL acts as a mediating variable, as shown by the SEM (Table 4), between the intermediate and direct determinants (notably, educational level and income), which influence both the mother’s OHL and her child’s oral health [18,34]. High maternal OHL appears to encourage preventive and educative behaviour beneficial to the development of children’s oral hygiene practices (brushing). We reported, confirming previous results, that mothers’ OHL is significantly linked to their oral hygiene practices [19] and to their children’s preventive behaviour (brushing) [18]. These result points in the same direction as those of several studies that consider that OHL’sassociation with oral health status is explained by its strengthening the knowledge, attitudes and practices that lead to good oral health. The basic knowledge required to prevent oral disease probably facilitates understanding of the recommendations given [18,22].

The level of OHL was low in slightly over half of the mothers in our study. A study using the same measurement tool (OHL-AQ) observed a better level of OHL in Indian women [35]. Other studies using the REALD-30 [20]found that “caregivers”, mainly women, had higher OHL levels in Hong Kong [29]and in North Carolina (USA) [8]. A study in New Zealand concluded that 37.6% of adults, mostly women, had a low OHL level [31]. The main explanation for these contrasting results is that these studies were performed in countries with high and average incomes, on heterogeneous samples, and with very different measurement tools. Many studies were limited to evaluating functional OHL (reading, calculation and understanding capacities) [20,36]. Other studies, such as those in New Zealand and India, tried to take into account the more specifically “oral health” aspects of OHL, that is, the sources of oral health information, oral health knowledge, communication skills, and appropriate decision-making [31,35].

This study has strengths and limitations. Its strengths stem from the method used to collect oral health data–clinical examination. The sample size is also a strong point, although its degree of homogeneity in a disadvantaged district lessened its ability to reveal the magnitude of the social gradient of health in Senegal, where few epidemiological oral health data are available. This work is one of the rare studies that have focused on the relation between the OHL of mothers and the oral health of their children, and it is the first of its kind in Senegal.

The limitation of this study concerns the measurement of OHL, whose translation into the local language was not validated. However, the chief investigator was fluent in the local language and knowledgeable about the country’s cultural context. In view of the limitations of the tools used to evaluate OHL in Senegal, it appears advisable to develop a measurement tool that takes better account of the importance of various modes of communication.

## Conclusion

This study provides important data, some of them original, on the individual and family determinants of the dental caries of the children in Pikine and on the effects of their mother’s OHL level. Mothers play a key role in all early and effective interventions concerning their children. Thus it is important to take OHL into account in developing and implementing a policy aimed at improving oral health and reducing its inequalities in Senegal.

## Appendix

### Model of study

A pathway model based on the WHO Commission on Social Determinants of Health (25) was used to model the mechanisms of social inequalities in oral health. It covers the complex relations between structural determinants, intermediary determinants, direct determinants (material conditions, psychosocial factors and oral health behaviour) and oral health.

Our study model was made operational via “proxy” variables that could be feasibly collected in a population-based survey. Thus the material conditions were represented by household wealth and density and the psychosocial factors by the presence of social support estimated by the frequency of contacts (family, friends). Since income is sensitive to information, wealth has been assessed by establishing the list of assets available to the household. This list has been compared to a list of basic goods defined by reference to the indicators used in poverty monitoring reports in Senegal. These properties are: accommodation with rooms equipped with bed or mattress, electric lighting, adequate sanitary facilities, source of drinking water, 3 daily meals, possession of television and / or radio (for information). Density is defined by the ratio of household members to living quarters. The mothers’ knowledge, attitudes and practices related to oral health were studied through three variables: the mothers’ oral health literacy (OHL), attitude to oral health, and practice of oral hygiene (tooth brushing).

### Methods

#### Sampling and sample size

Two-stage cluster sampling was performed: the municipality and the household. In the first stage, 9 municipalities out of 16 were drawn by lot. For the second stage, two households were chosen at random in each building or concession and 2 children at most in each family.

We used the so-called “exploratory” method defined by WHO [30]to determine the number of subjects required. It suggests selecting between 20 and 50 subjects per site as a function of the prevalence of the disease. A prevalence of 68% of dental caries in the children of Pikine [7] led to estimating the number at 35 subjects. The size of the sample was therefore (N = 35x9 = 315) 315 children and as many mothers (315).

#### Household variables

Household wealth was defined with reference to a list of household goods. This list was compared to a list of basic goods defined with reference to indicators used in reports monitoring poverty in Senegal. It permits classifying the households in 3 categories: poorest, poor, least poor. The density of dwelling occupancy was defined by the number of household members divided by the number of rooms in the dwelling. This variable was defined in two classes (less dense, denseras a function of the median of the distribution).

#### Oral health literacy: OHL-AQ questionnaire

This contains 17 items distributed in 4 dimensions: Reading and comprehension; Comprehension of numbers; Listening, comprehension and communication and Appropriate decision-making relating to oral health. Each correct response was given a score of 1 and a wrong response a score of 0.

#### Composite variable

This is a quantitative variable that was used in the “path analysis” type structural equation model (SEM) to satisfy the need to understand, through linear logic, the relations between the socioeconomic characteristics (or social position) of mothers, their OHL level and the dental caries of children. It is composed of three variables: mother’s educational level, her occupation, and household dwelling density. Scores are assigned to the different categories of variables and the mean scores are calculated.

#### Plan of analysis

Two logistic regression models and a structural equation model (SEM) were built. The first tested the links between dental caries, OHL and the social position factors. The second model had two levels: the first tested the factors associated with dental caries, the OHL level, and the other direct determinants (household wealth and social support);the second level replaced the OHL level by the mother’s oral hygiene practice in order to introduce a “knowledge, attitude, practice” variable. The structural equation model (SEM) was also used to test the structure of the relations between the explanatory variables and dental caries since these types of linear model are best suited for structural analysis. To do this, two hypotheses of causal chains were tested: the socioeconomic characteristics of mothers and households were linked to OHL; then children’s dental caries was linked to the mothers’ OHL. The quality (of adjustment) of the SEM model was evaluated with the Root Mean Squared Error of Approximation (RMSEA) and the Chi-2 test, while the Hosmer-Lemeshowtest was used to test the goodness of fit of the logistical models.
